# Mitochondrial DNA Mutations in Cancer

**DOI:** 10.1371/journal.pmed.0020401

**Published:** 2005-11-29

**Authors:** Stefanie Zanssen, Eric A Schon

## Abstract

One can no longer ignore mitochondria in cancer biology, argue Zanssen and Schon.

In 1956, the German biochemist and Nobel Laureate Otto Warburg proposed that cancer is caused by altered metabolism and by deranged energy processing in mitochondria [1]. After falling on deaf ears for decades, his theory has recently enjoyed a resurrection, coinciding with an explosion of new information on the role of mitochondria in energy metabolism and oxygen sensing.

## Mitochondrial Enzyme Deficiencies in Inherited Neoplasia

**Figure 1:**
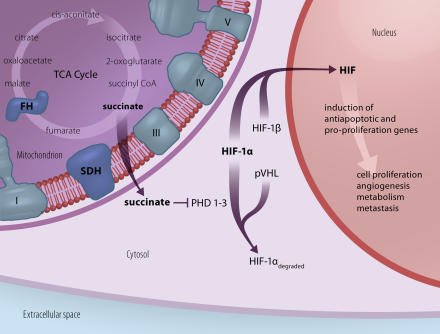
Schematic Model for Tumorigenesis in SDH deficiency SDH is a component of the OxPhos complexes (I–IV) as well as the TCA cycle. Due to SDH deficiency succinate accumulates intramitochondrially (the same occurs when FH is deficient) and is transported into the cytosol. Here, its elevated concentration inhibits prolyl hydroxylases (PDH1-3), which can no longer degrade HIF-1α even under normoxic conditions. Heterodimerisation of stabilized HIF-1α and HIF-1β now forms HIF, which induces transcription of nuclear genes involved in tumor progression [5]. (Illustration: Giovanni Maki)

Mitochondrial defects have been associated with severe neurodegenerative disorders [2], and, more recently, with primary hereditary neoplasias. Germline heterozygote mutations in the nucleus-encoded mitochondrial succinate dehydrogenase (SDH) subunits—a tricarboxylic acid cycle (TCA) enzyme, which is also part of the respiratory chain—cause inherited pheochromocytomas and paragangliomas. Mutations in another TCA enzyme, fumarate hydratase (FH), cause cutaneous and uterine leiomyomas, as well as renal cell carcinomas [3]. Mitochondrial proteins may also play a role in the development of the sporadic kidney tumor oncocytoma [4]. Selak et al. has recently shed light on the mysterious connection between tumors and SDH/FH [5]; they showed that the hypoxia-inducible factor (HIF)–mediated signaling pathway, known to be tumorigenic in von Hippel Lindau syndrome, also plays a role in SDH and FH deficiency (Figure 1) [5].

## Somatic mtDNA Mutations in Sporadic Tumors

Mitochondrial DNA (mtDNA) mutations have also been linked to nonhereditary tumors. Intragenic deletions (see Glossary) [6], missense and chain-terminating point mutations [7], and alterations of homopolymeric sequences [8] have been identified in nearly every type of tumor. Mutations have been described in the hypervariable regions of the mitochondrial D-loop, the section of DNA controlling mtDNA transcription and replication that is most prone to mutation. Mutations have also been described in all 22 tRNAs, both rRNAs, and all 13 of the mtDNA-encoded subunits of the respiratory chain complexes.

**Glossary**

**Chain-terminating point mutation:** A point mutation that leads to a premature stop codon

**D-loop analysis:** Analysis of polymorphisms in the mtDNA control region, known as the D-loop

**Ethnic-specific haplotype-defining polymorphism:** A polymorphism representing an mtDNA variant found exclusively in a specific population. Haplotypes H, K, J, and U are found in European populations

**Homopolymeric sequences:** Consecutive runs of the same nucleotide

**Intragenic deletion:** Loss of a contiguous segment of DNA from a gene

**Missense mutation:** A point mutation in a codon resulting in the alteration of one encoded amino acid to another

**Mitomap database:** An online human mitochondrial genome database (http://www.mitomap.org/)

**Nuclear mitochondrial pseudogenes:** Nuclear DNA sequences homologous to mtDNA sequences, but without any function

**ROS:** Reactive oxygen species

**rRNA:** Ribosomal RNA

**tRNA:** Transfer RNA

These findings pose a key conceptual question: how do these mutations arise and then establish themselves so quickly in the tumor? Many of the (noninherited) mtDNA mutations lead to the mtDNA of the tumor becoming heteroplasmic; i.e., they are a mixture of normal germline mtDNAs found in the noncancerous tissue and mtDNAs containing one or more mutations. Even more surprising than this heteroplasmy is the observation that most mutations are apparently homoplasmic (i.e., there is no evidence of the germline mtDNA haplotype in the tumor), and further, some of those genomes contain not one, but two, three, and even four mutations, all on the same mtDNA circle. How can a cell with mitochondrial haplotype A in noncancerous tissue shift its mitochondrial genotype to haplotype B so rapidly in the adjacent tumor? Mathematical models of heteroplasmic shifting in rapidly dividing tumor cells [9] have been invoked to explain this conundrum, but the mechanism of shifting remains both elusive and controversial.

In this issue of *PLoS Medicine*, Salas et al. question the very foundation of the observation of haplotype shifting in tumors [10]. They present evidence that much of the literature demonstrating mutations—whether heteroplasmic or homoplasmic—has been laden with technical and conceptual errors that reduce the finding of mtDNA mutations in tumors to the level of technical artifact.

Salas et al. rightly emphasize the importance of the highest standards in mtDNA analysis to produce reliable results in the many investigations on this subject [10]. In summary, they point out that double-checking to avoid sample mix-ups and using stringent procedures to extract either purified mtDNA or total DNA containing the mtDNA will guarantee high-quality templates (avoiding, for example, amplification of nuclear mitochondrial pseudogenes, as in the criticized study of Reddy et al. [11]). They also point out that controls should be ethnicity- and age-matched, and that every mutation should be checked to see whether the mutation is listed in the Mitomap database or is an ethnic-specific haplotype-defining polymorphism. Furthermore, when interpreting results, researchers should assess whether the missense mutations that are being implicated in carcinogenesis fulfill the criteria for pathogenicity (for example, they should occur in highly conserved regions or destroy structurally important motifs). If these criteria are adhered to, Salas et al. claim that the vast majority of mtDNA mutations in tumors will simply disappear [10].

However, although technical errors probably do account for some of the positives found in some of the literature, they cannot account for all of them. Putting aside the issue of mechanism, the question that remains—can mtDNA haplotypes shift rapidly in tumors—is not only still with us, but is almost certainly answered affirmatively.

**Figure 2:**
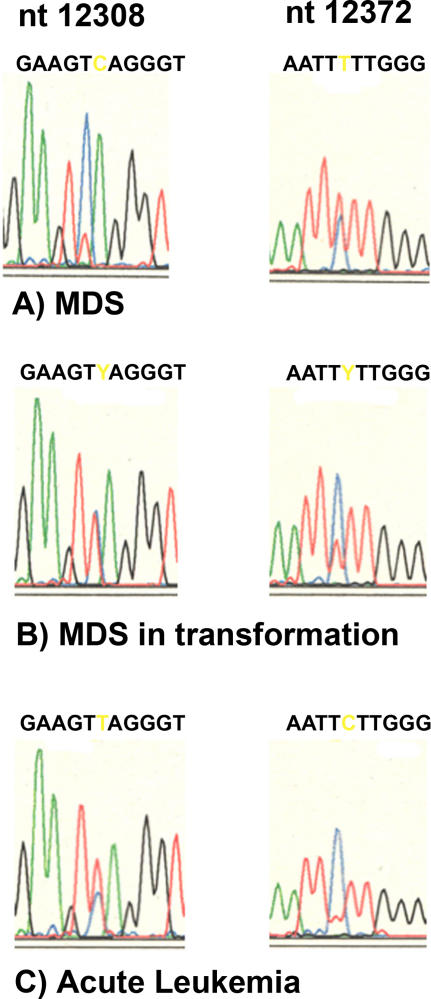
Mutations *C12308T* and *T12372C* Alter Polymorphisms Specifying European Mitochondrial Haplotype U Mutation load in (A) MDS, (B) MDS in transformation, and (C) acute leukemia.

We would interpret the mtDNA sequencing data from the phylogenetic point of view differently than Salas and colleagues. mtDNA haplotype-specifying polymorphisms [12] arising on a background of a fixed mitochondrial germline haplotype are seen commonly in tumors, for example, in a patient with myelodysplastic syndrome ([MDS], case one in [13]) who had mtDNA germline haplotype U (confirmed by U-specifying polymorphisms at nt 150, 3197, 12308, and 12372). Such polymorphisms are not necessarily due to sequencing errors or contaminations. The typical slow progression of MDS to acute leukemia in this patient made it possible to monitor the shift from “initial” germline homoplasmy to heteroplasmy to “final” homoplasmy of mutated mtDNA in the tumor in different stages of the disease. Figure 2 shows that the haplotype U–specifying positions 12308 and 12372 in the patient's platelets had a “beginning” heteroplasmy in MDS (Figure 2A), which reached more than 50% when the cells were in transformation (Figure 2B), and “ended” at homoplasmy when the acute leukemia arose (Figure 2C). Sample contamination or a shift from haplotype U to H can be excluded here because all other haplotype-specific polymorphisms were homoplasmic for type U, not H. The same observation was made in patient 2 of the cited study from Kirches et al. [14]. This patient had a germline mitochondrial haplotype J, which “shifted” in positions 185, 295, and 16126 back to the phylogenetically older haplotype H, but shifted in position 195 to haplotype W and in position 204 to nowhere.

Although the mechanism of rapid shifting is still unclear, random processes, extensively modeled in computer systems [9], may well explain the presence of homoplasmic mutations in cancer. It may be that certain mitochondrial haplotypes or pathogenic mutations represent a selective advantage in tumor progression, similar to the overrepresentation of haplotype J seen in Leber hereditary optic neuropathy, a maternally inherited mitochondrial disease [15].

## Challenges for the Future

Warburg was right. One can no longer ignore mitochondria in cancer biology. Mutations of mtDNA, even driven by random processes during malignant transformation, present an excellent possibility for early tumor detection using, e.g., D-loop analysis of bodily fluids from patients with tumors [16]. (As an aside, direct sequencing of tumor mtDNA, as performed by many groups, is a poor screening technique, as it misses levels of heteroplasmy below approximately 20%; a better method is denaturing high-performance liquid chromatography followed by confirmatory polymerase chain reaction/restriction fragment length polymorphism analysis.)

The proposal that mtDNA mutations and respiratory dysfunction may be linked directly to carcinogenesis via apoptotic or reactive oxygen species (ROS)–mediated pathways is challenging, but urgently needs experimental proof. Questions regarding whether the HIF-mediated pathway is also initiated in hypoxia and mitochondrial deficiency [17], both characteristics of tumors, also need to be clarified. If these pathways are confirmed as being involved in tumorigenesis, metabolic targeting (e.g., blocking the HIF-pathway by administration of α-ketoglutarate) of the mitochondrion in cancer may open up new avenues to antineoplastic therapies and prevention of cancer.

## Abbreviations

FH: fumarate hydratase
HIF: hypoxia-inducible factor
MDS: myelodysplastic syndrome
mtDNA: mitochondrial DNA
SDH: succinate dehydrogenase
TCA: tricarboxylic acid cycle
